# Retention of 3D-printed versus conventional metal-reinforced acrylic mandibular denture bases: a comparative study

**DOI:** 10.1186/s12903-025-07188-4

**Published:** 2025-11-28

**Authors:** Marwa E El-Din, Ayman F. Elawady, Hager F. Elsadany

**Affiliations:** 1 Prosthodontics Department, Faculty of Dentistry, Misr University for Science & Technology, Giza, Egypt; 2 Prosthodontics Department, Oral and Dental Research Institution, National Research Centre, Cairo, Egypt; 3 Prosthodontics Department, Faculty of Dental Medicine for Girls, Al Azhar University, Cairo, Egypt

**Keywords:** Complete denture, Metal reinforced denture, Retention, 3D-printing

## Abstract

**Objective:**

To evaluate the retention of complete mandibular dentures produced via 3D-printing in comparison to those constructed from metal-reinforced acrylic resin regarding their retention.

**Materials and methods:**

This in vivo study involved the selection of twenty patients who were completely edentulous from the outpatient clinic of the Removable Prosthodontics Department at Misr University of Science and Technology. The participants were divided into two groups, each containing an equal number of patients; the first group received complete dentures produced via 3D printing technology. For the second patient group, metal-reinforced acrylic resin was used to make mandibular dentures, while conventional maxillary dentures were constructed. The evaluation of retention was carried out for both groups.

**Results:**

A study of various types of dentures revealed that 3D-printed dentures exhibited a statistically significant improvement in retention (*P* < 0.05) relative to acrylic resin dentures that were reinforced with metal. Moreover, pairwise comparisons revealed significant increases in mean retention values at the one-month and three-month follow-up assessments.

**Conclusion:**

The results of this clinical study led to the following conclusions: Compared to complete denture bases manufactured via conventional manufacturing techniques, those fabricated via 3D-printing offer superior fit and retention. The retention of dentures, irrespective of their type, tends to improve over time.

## Introduction

As a result of aging populations in developed nations and subpar dental treatment in underdeveloped nations, edentulism is a serious public health concern. Patients with no teeth have a biased quality of life and dietary intake because of their condition. Over the coming decades, complete dentures (CDs) are expected to rise steadily, notwithstanding the anticipated decline in age-specific rates of edentulism [1]. Edentulism is recognized by the World Health Organization (WHO) [2] as a physical disability. Entirely edentulous individuals frequently choose CDs as their treatment method. While CDs offer several advantages, many patients have expressed concerns regarding the loss of stability and retention, which can detrimentally influence their quality of life and level of satisfaction.

The mandibular CD is more likely to have retention issues than the maxillary CD is for a variety of reasons, including the former’s smaller supporting structure relative to the maxillary arch, the latter’s strong tongue muscles, and the fact that each step of denture construction should be given careful consideration. The proximal mucosal-denture base contact achieves beneficial retention [3].

When it comes to making dentures, polymethyl methacrylate (PMMA) is the preferred material. There is dimensional shrinkage of the resin throughout the polymerization process. The rise of the denture base away from the posterior palate during polymerization is due to shrinkage, which is brought on by disparities in the densities of the monomer and the polymer making contact [4].

Denture quality might be enhanced by the application of computer-aided design and computer-aided manufacturing technology (CAD-CAM), which also offers the ability to shorten turnaround times and simplify complicated laboratory processes. CDs can be made via additive three-dimensional 3D-printing, also known as rapid prototyping (RP), or subtractive computerized numerical control (CNC) machining [5–7].

CAD ensures that the denture base thickness is uniform and can be conveniently estimated. Moreover, the existence of digital data enables the after-time production of dentures in the event of loss or damage [8]. The retention of milled dentures from pre-polymerized PMMA is preferred over their conventional heat-polymerized acrylic resin counterparts due to less polymerization shrinkage, resulting in superior adaptation [6].

Metal alloys have better mechanical and physical qualities than PMMA. They provide a more robust solution since they are stronger, have better fatigue, fracture strengths, and can be cast in thin sections without compromising their rigidity or fracture resistance. This savor, together with its high thermal conductivity, furnishes patients with a more natural “feel” of the prosthesis [9].

In addition to being dimensionally robust and biocompatible, metallic denture bases have a highly polished surface that makes hygiene care easier. They help when bruxism or single maxillary dentures are present, and the denture base must be strengthened. They are beneficial in cases with bruxism or single maxillary dentures, which require strengthening of the denture base [10].

The null hypothesis assumes that there will be no significant differences between the two groups regarding retention.

## Materials and methods

From the outpatient clinics of the College of Oral and Dental Surgery at Al Azhar University and the Medical Excellence Centre of the National Research Centre in Cairo, Egypt. (Clinical trial registration number: (NCT07061860) (07/11/2025). https://clinicaltrials.gov/study/NCT07061860. Twenty completely edentulous patients were chosen. The study included patients who were aged between 55 and 60 years and had been completely edentulous for at least one year. All procedures were explained to all patients, and informed written consent was obtained [11]. Inclusion and exclusion criteria for the patients. Table (1).

**Table 1:** Patient inclusion and exclusion criteria

Included patient criteria	Excluded patient criteria
Class I maxillary-mandibular relationship	smokers
well-maintained oral mucosa	any abnormalities in hard or soft tissues
normal salivary flow	severe ridge undercut
	received radiation therapy to the head and neck region

A total of twenty patients were single-blinded and randomly divided into two groups, with each group containing ten participants. CDs constructed from PMMA were used to create the mandibular and maxillary 3D-printed dentures that were given to patients in Group I. Group II patients received CDs; mandibular dentures were constructed from polymethyl methacrylate reinforced by a metal framework, and maxillary dentures were constructed from conventional PMMA resin. Retention for both groups was assessed at the point of insertion as well as at three- and six-month post-insertion, utilizing a retention force gauge device.

### Sample size calculation

Based on previous studies by Mohamed GF [12] and Elmorsy et al. [13] the outcome variable is normally distributed, retention is measured by a digital force meter, and the measuring unit is Newton. The samples were divided into two equal groups. The first group of patients administered CDs created through 3D-printing. The second group of patients also received conventional maxillary dentures, while mandibular dentures were fabricated with metal-reinforced acrylic resin; an effect size of approximately 0.4 is expected. A total sample size of 12 will be sufficient with a power of 80% and 5% significance level. This number will be increased to 20 (10 in each of the two groups) to compensate for follow-up losses. The sample size was determined through the application of the G Power program.

### Denture construction

#### Construction of the 3D model

**Fig. 1:**
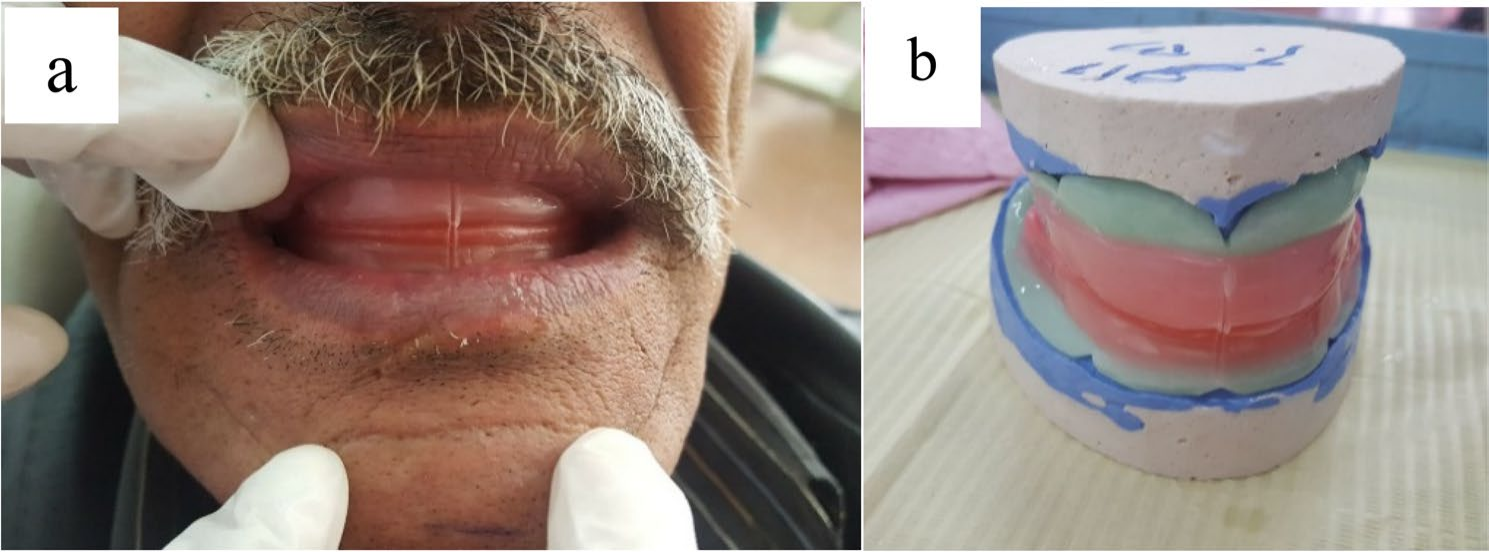
**a**, **b** Jaw relation record

Under the basic protocol for CDs, irreversible hydrocolloid impression material (Cavex alginate, Cavex, Holland) was used to make primary impressions. The borders were effectively shaped using a green stick impression material. (Impression Compound; Kerr Corp.), and subsequently, a secondary impression was made with a zinc oxide-eugenol (SS White, England)-based impression paste. A face bow was used to ensure the correct alignment of the maxillary cast on the articulator device.The wax wafer method was used to record the centric relationship at the appropriate vertical dimension of occlusion by placing the maxillary and mandibular occlusion blocks into the patient’s mouth and adding two layers of softened wax.The patient was instructed to retrude his or her mandible and close in the most retruded position. The mandibular cast was then placed on the basis of the centric jaw relationship. (Fig. 1a, b).

**Fig. 2:**
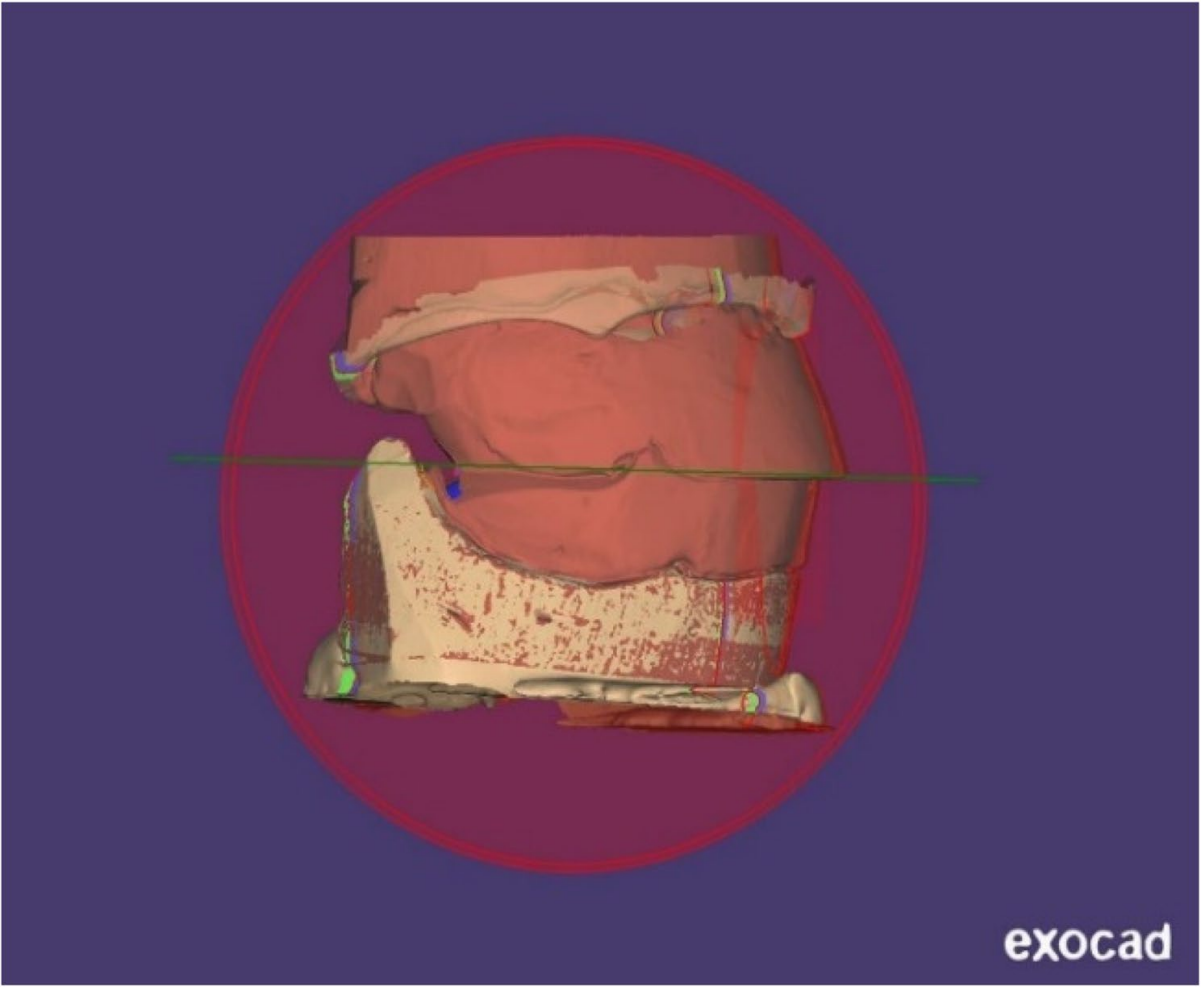
Scanning occlusion blocks

**Fig. 3:**
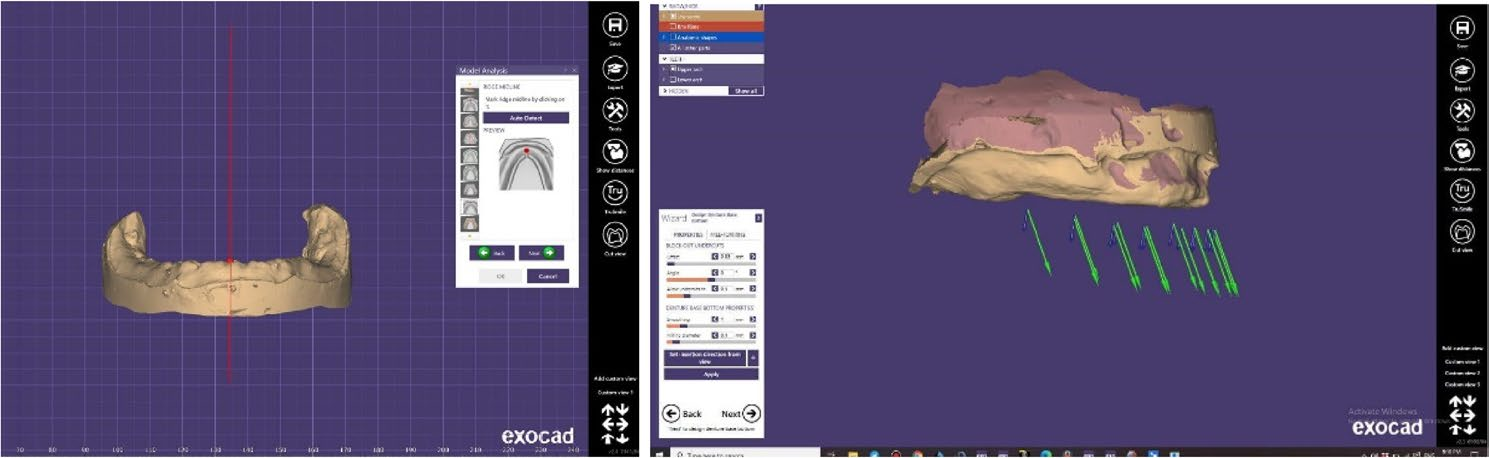
Analyzing and designing the dentures

For the fabrication of a 3D-printed denture, the procedure was as follows for each patient:

a Earned data: Master castings and upper and lower secondary impressions were scanned via an extra-oral scanner (Siemens INEOS X5 scanner from Germany) to obtain the data. The jaw-relationship records were put on their master cast and covered with scanner spray (Bilkim 3D scanning spray Menemen, Izmir, Turkey) before being scanned via the previously stated scanner. (Fig. 2)
b Designing procedures: CAD software (Exocad software, Exocad GmbH, Darmstadt, Germany) was used to import files in the standard tessellation language (STL) formats of secondary impressions, master castings, and jaw-relationship information. The software essentially made simultaneous mounting and alignment practical. This makes it possible to finish more analysis and design. Using a 3D-printer (photon S, Anycubic, China), two STL files, one for the denture base and one for the teeth, were manufactured independently for each denture. The clinician was given the developed dentures for approval. (Fig. 3)

**Fig. 4:**
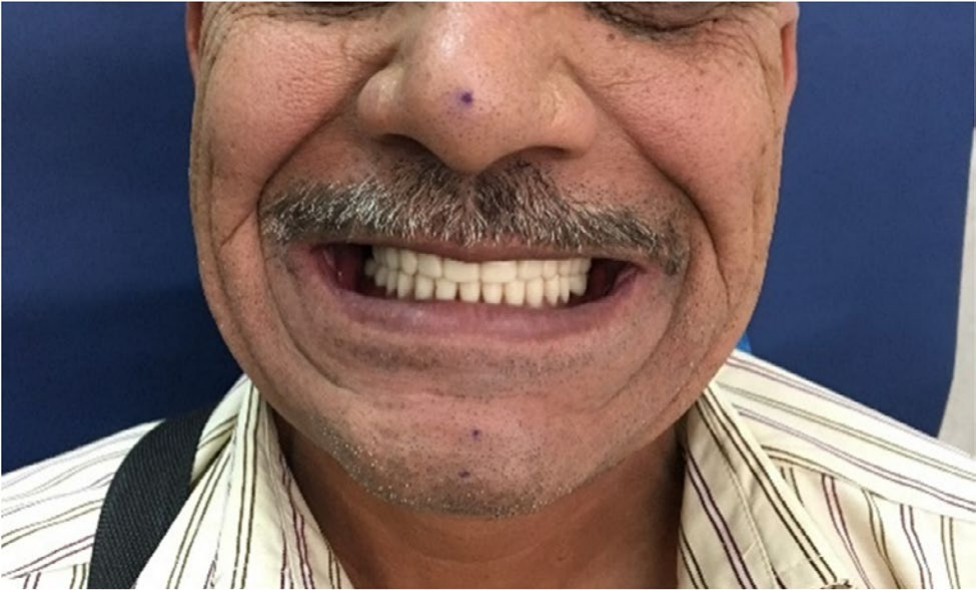
3-D complete denture

Fabrication procedures: The 3D-printing software received the STL files for the planned dentures. Using a desktop 3D-printer, Stereolithography apparatus (SLA) technology was used to fabricate the dentures. While teeth were printed (NextDent CDOWNTEC, NextDent B.V., Soesterberg, Netherlands), denture bases were printed using pink denture base resin (NextDent Denture 3D+, NextDent B.V., Soesterberg, Netherlands). Self-curing acrylic resin was used to attach artificial acrylic teeth to the denture base. To accelerate the polymerization process, the denture bases were post-cured and ultrasonically cleaned with alcohol for five minutes after the printing step. To test retention, stability, and occlusion, the completed dentures were finaly inserted into the patient’s mouth. (Fig. 4).

#### Construction of metal-reinforced complete dentures

Irreversible hydrocolloid impression material (Cavex alginate, Cavex, Holland) was used to make primary impressions. The borders were molded functionally with an impression compound (Kerr Corp.; green stick impression compound) (impression compound; Kerr Corp). A secondary impression was obtained utilizing a zinc oxide-eugenol (SS White, England)- based impression paste. The mandibular master cast with the wax spacer was duplicated for metal base fabrication. After this procedure, a refractory copy of the modified master cast was provided.

**Fig. 5:**
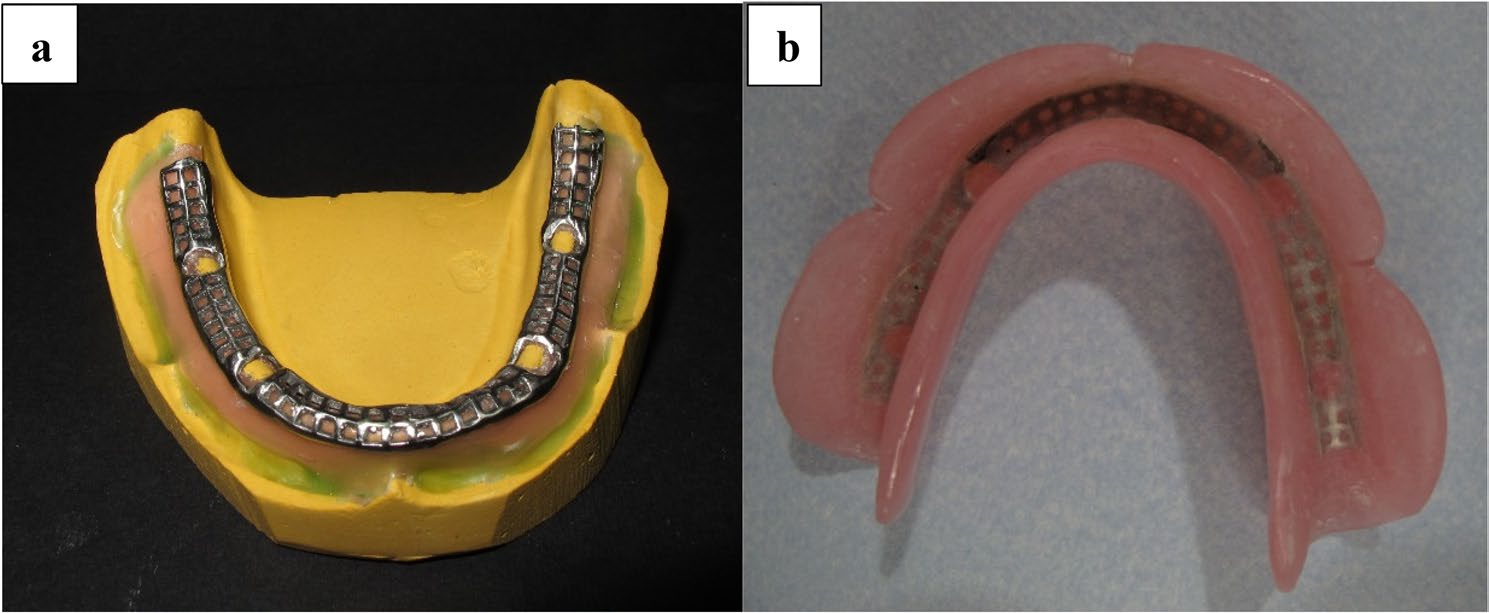
**a** Metal framework. **b** Final denture

Metal framework: The modeling process involved the use of a wax profile, which was strategically placed on the crest and convexity of a residual ridge on the refractory cast. This was followed by the steps of spruing, investing, burnout, and subsequent casting of the metal framework with a cobalt-chrome-molybdenum (Co-Cr-Mo) alloy. (Fig. 5.a).

The jaw and facebow relationships were recorded. For the best feasible aesthetics and lip support, the smile line, canine, midline, and anterior tooth locations were determined. The models for the metal frame within the mandibular denture were waxed up, cast, and finished after the prosthetic teeth were placed and tested. Denture polishing and finishing were completed. (Fig. 5.b). Finally, the denture was inserted after being inspected for occlusion, stability, retention, and extension.

#### Retention measurements

To assess retention, a digital force gauge (Digital Force Gauge EXTECH 475044) was employed. All of the mandibular dentures were modified to receive a custom-made metal framework consisting of three tubes connected by a hook at the determining geometric center of the denture, two tubes extended 2 cm above the occlusal plane from the retromolar pad region, and one was made at the midline region lingual and below the central incisors. The polished surface of the mandibular denture was then joined to the specially constructed custom-made metal framework using self-curing acrylic resin.

#### The retention of mandibular dentures was estimated as follows

**Fig. 6:**
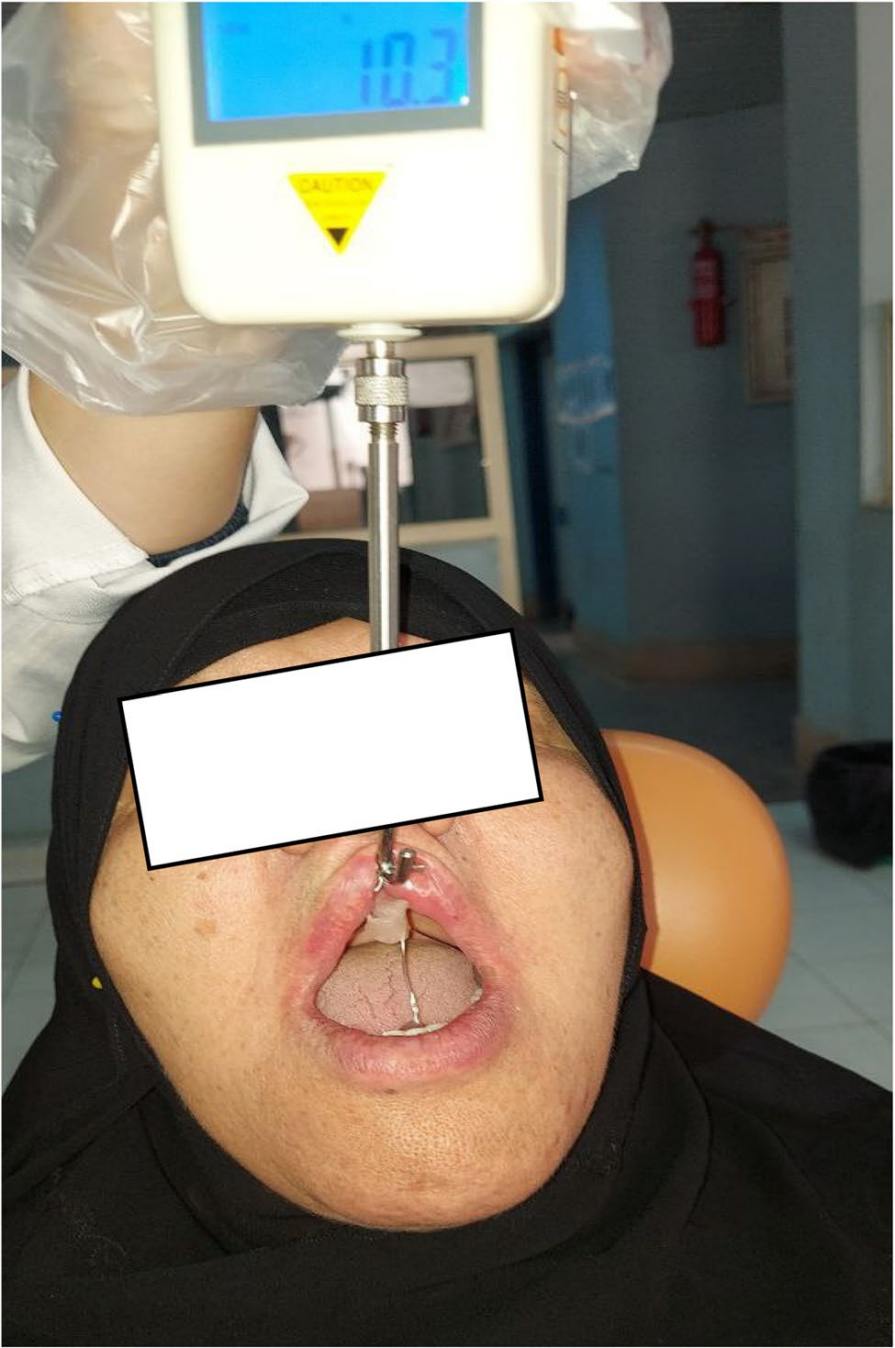
Retention measurements

Following the insertion of the dentures, a metallic probe from the digital force meter was connected to the metal hook positioned at the geometric center of the mandibular dentures. A vertical pulling force was then applied to assess the retention of the dentures. The dynamometer recorded the peak force and constantly displayed the dislodging forces in Newtons. All the measurements were taken with a 1-minute resting time between each measurement. All measurements were conducted by a single operator. (Fig. 6)

### Statistical analysis

The mean and standard deviation were used to present all of the data. There were two tables and two figures with the data. GraphPad Prism, Windows Excel, and SPSS 16 ^®^ (Statistical Package for Scientific Studies) were used for statistical analysis.

The provided data were examined for normality using the Shapiro-Wilk and Kolmogorov-Smirnov tests. The findings demonstrated that the significance level (*P*-value > 0.05) was insignificant and that all of the data were normally distributed (parametric data) and resembled a normal bell curve.

Therefore, groups were compared using an independent t-test, intervals were compared using a one-way ANOVA test, and multiple comparisons were handled using Tukey’s Post Hoc test.

### Effect of time (Table 2; Fig. 7)

In group I, there was a significant difference between all intervals as *P* < 0.05. At delivery was significantly the lowest retention (18.49 ± 0.52), then after 3 months (20.51 ± 0.58), while after 6 months (21.77 ± 0.59) was significantly the highest, with 20.00% increase in retention for 6 months. 

There was a significant difference (*P* < 0.05) between all intervals in group II, at delivery was significantly the lowest retention (12.56 ± 0.31), while there was an insignificant difference between after 3 months (13.82 ± 0.56) and after 6 months (14.19 ± 0.49), with 15.9% increase in retention for 6 months. 

**Table 2:** The mean and standard deviation of retention for both groups, along with a comparison of various intervals and the percentage change observed in each group

	Follow up	M	SD	% change	*P* value
Group I (3D printed denture)	**At delivery**	18.49 ^**a**^	0.52	20.00%	< 0.001*
	**After 3 months**	20.51 ^**b**^	0.58		
	**After 6 months**	21.77 ^**c**^	0.59		
Group II (metal reinforced denture)	**At delivery**	12.56 ^**a**^	0.31	15.90%	< 0.001*
	**After 3 months**	13.82 ^**b**^	0.56		
	**After 6 months**	14.19 ^**b**^	0.49		

Means that are represented by varying superscript letters showed statistically significant differences, as indicated by a *p*-value of less than 0.05. Means marked with the same superscript letters showed no significant differences, as *P* values were greater than 0.05

*M* mean, *SD* standard deviation

*Significant difference at *P* <0.05

**Fig. 7:**
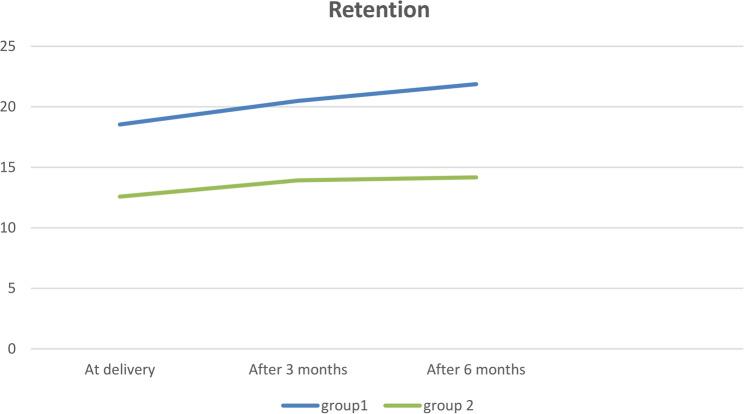
Line chart illustrating the variations in retention rates for both groups across various time intervals

### Effects of different construction techniques (Table 3; Fig. 8)

A comparison between groups I and II revealed that group II was significantly higher than group I at all intervals, as *P* < 0.05.

**Table 3:** Comparison was conducted to compare the retention rates between Group I and Group II at various intervals, utilizing the Independent t-test for statistical evaluation

Interval	Group I		Group II		**P value**	**MD ± SEM**
	**M**	**SD**	**M**	**SD**		
At time delivery	18.49	0.52	12.56	0.31	0.001 *	5.9 ± 0.2
After 3 months	20.51	0.58	13.82	0.56	0.001 *	6.6 ± 0.3
After 6 months	21.77	0.5	14.19	0.49	0.001 *	6.63 ± 0.3

Difference between means (B - A) ± standard error of the mean

*M* mean, *SD* standard deviation

*Significant difference at P <0.05

**Fig. 8:**
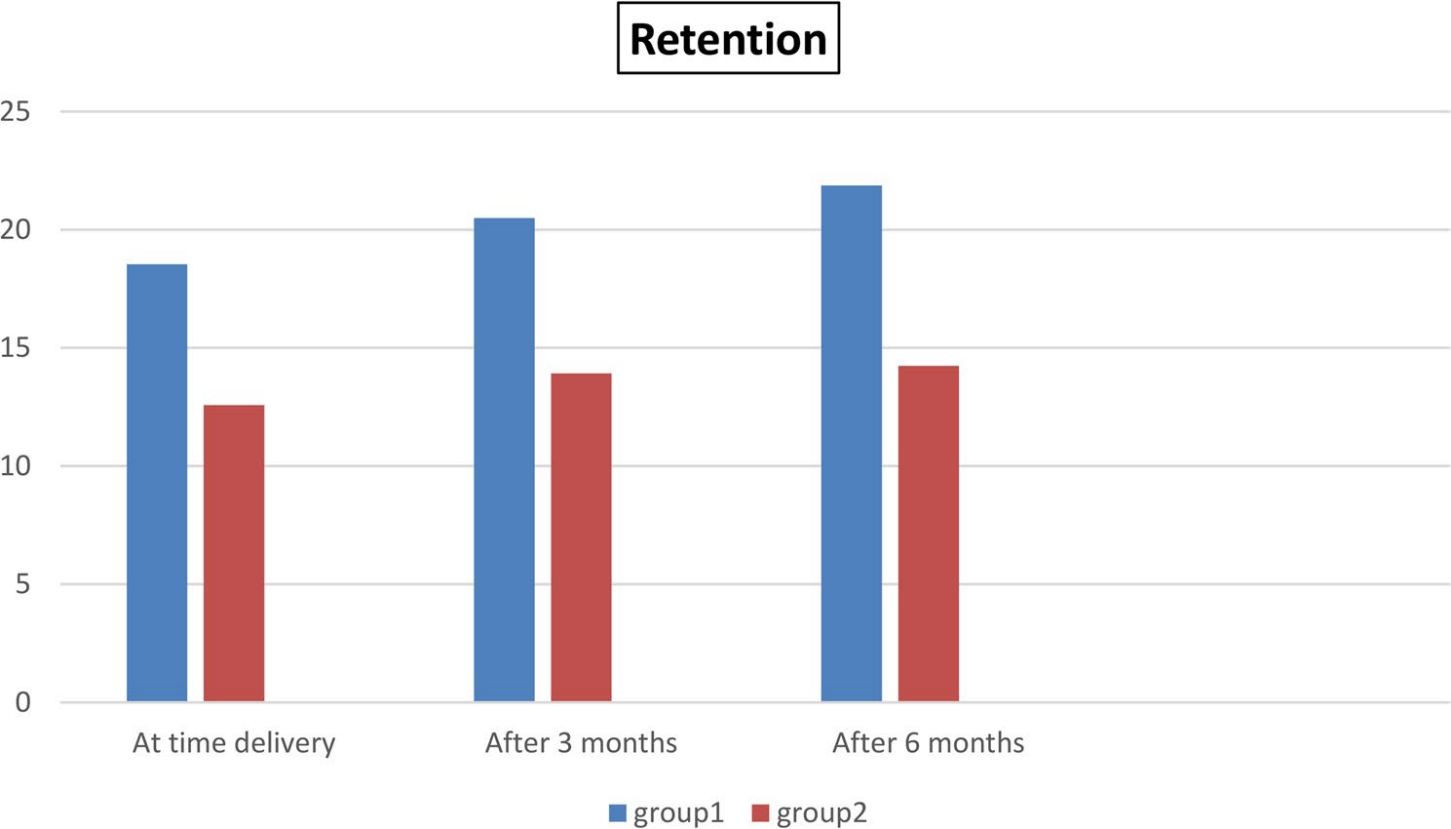
Bar chart representing retention in both groups

## Discussion

Retention, stability, and support are the three main components that make full dentures successful. Support prolongs the life of the entire denture, while retention and stability provide patients with psychological comfort. Border molding, which is required to keep the denture borders in contact with surrounding vestibular tissues both during rest and during functional activities, can help accomplish retention. Zinc-oxide eugenol impression material can provide excellent retention values [14] and a good flow over peripheral surfaces when used as the final imprint material.

There are various methods for obtaining jaw-relationship records and secondary impressions. Improvements that result in the manual recording of the maxillomandibular connection may make digital CD systems more appealing; Schwindling et al. When evaluating denture bases, those fabricated through 3D-printing or milling from traditional impressions demonstrate a markedly greater retentive force than those created from intraoral scans [15–20].

Because there is no polymerization shrinkage, a finer fit could account for the extraordinary retention of the CDs and amazing suction effect [21]. The production process can enhance the authenticity of adaptations and the reliability of digital CDs, provided that the physical properties of the resins do not substantially influence the retention of CAD/CAM dentures. However, the excellent manufacturing technique of these resins encourages digital CD adherence and adaptation, and tests have demonstrated that digital CD retention outperforms that of traditional prosthesis over time [22].

Multiple studies found that digital complete dentures are relatively superior to conventional complete dentures in terms of retention over time, as denture base retention depends on multiple factors, including adaptation, dimensional changes, and accuracy of construction technique [23].

In contrast to the results of this study, another in vitro study evaluated denture base adaptation of digitally and conventionally fabricated dentures, exhibiting superiority of denture adaptation of conventional dentures over digitally fabricated dentures [24]. Because the 3D-printing technique relies on utilization of unpolymerized resins for manufacturing complete dentures. Upon denture processing, final light-polymerization step was mandated to fulfill the process, thus, polymerization shrinkage became theoretically possible throughout the workflow [25].

The utilization of metal framework reinforcement is a widely adopted method in the production of complete dentures, primarily because it significantly improves the overall quality of the prosthetic device. This technique is esteemed for its contributions to enhancing fracture resistance, achieving dimensional stability, improving accuracy, decreasing weight, and facilitating the retention of the final prosthesis. Balch et al. [26] suggested that the addition of a metal framework to polymethyl methacrylate positively influences its ability to withstand fractures. The high degree of malleability, combined with the superior strength of metal, allows it to function effectively as a structural support for the acrylic component in denture fabrication. Consequently, this contributes to the ability to withstand flexural fatigue and stress concentration, thus enhancing the strength of the denture and increasing its overall longevity [27, 28].

### Limitations

The study’s findings are based on a sample of only 20 participants. While our results show a promising trend, the small sample size limits our ability to make definitive conclusions about the intervention’s effectiveness. Future research with a larger sample is needed to confirm these findings. Further clinical studies over a longer time period are recommended to test the retention and patient satisfaction of 3D-printed dentures compared to conventional heat-cured acrylic dentures.

## Conclusion

From the findings of this clinical study, the following conclusions can be drawn: Complete denture bases fabricated via 3D-printing demonstrated a higher level of retention and fit than conventionally manufactured ones. Additionally, 3D-printed dentures often provide superior initial retention over time compared with their conventional counterparts.

## Data Availability

All data are available upon request from the corresponding author.

## Abbreviations

3D: Three-dimensional
PMMA: Polymethyl methacrylate
CAD-CAM: Computer-Aided Designed and Computer-Aided Manufacturing Technology
RP: Rapid Prototyping
CNC: Computerized Numerical Control
SLA: Stereolithography Apparatus
STL: Standard Triangle Language
Co-Cr-Mo: Cobalt-Chrome-Molybdenum Alloy
CD: Complete Denture

## References

[1] Han W, Li Y, Zhang Y, Hu P, Liu H, Ma Z, et al. Design and fabrication of complete dentures using CAD/CAM technology. Medicine. 2017;96:5435. 10.1097/MD.0000000000005435 PMC5228646 28072686

[2] Winkel T, Heijens L, Listl S, Gert Meijer G. What is the evidence on the added value of implant-supported overdentures? A review. Clin Implant Dent Relat Res. 2021;23:644–56. 34268866 10.1111/cid.13027 PMC8457103

[3] Ereifej NS, Oweis YG, Abu-Awwad M. The effect of using denture adhesives on patient satisfaction with complete dentures; a randomized clinical trial. BMC Oral Health. 2023;23(1):1027. 38114958 10.1186/s12903-023-03757-7 PMC10731830

[4] Kumar C, Surapaneni H, Ravikiran V, Chandra S, Balu S, Reddy N. Retention of denture bases fabricated by three different processing techniques - An *in vivo* study. J Int Soc Prev Community Dent. 2016;6(3):245–50. 27382542 10.4103/2231-0762.183106 PMC4916800

[5] Amin M, Ezzat M, Galaleldin Y. A comparison of denture base retention and adaptation between CAD- CAM and conventional fabrication techniques. Int J Prosthodont. 2023;36(4):469–478 . 10.11607/ijp.7193 37699188

[6] AlHelal A, AlRumaih HS, Kattadiyil MT, Baba NZ, Goodacre CJ. Comparison of retention between maxillary milled and conventional denture bases: a clinical study. J Prosthet Dent. 2017;117(2):233–8. 27765399 10.1016/j.prosdent.2016.08.007

[7] Yilmaz B, Azak AN, Alp G, Ekşi H. Use of CAD-CAM technology for the fabrication of complete dentures: an alternative technique. J Prosthet Dent. 2017;118(2):140–3. 28159344 10.1016/j.prosdent.2016.10.016

[8] Kattadiyil MT, Jekki R, Goodacre CJ, Baba NZ. Comparison of treatment outcomes in digital and conventional complete removable denture prosthesis fabrications in a predoctoral setting. J Prosthet Dent. 2015;114(6):818–25. 26412000 10.1016/j.prosdent.2015.08.001

[9] Chopra D, Gupta V, Sethi P. Maxillary complete denture with metal palate base: a case report. J Dent Sci Oral Rehabil. 2013;4:44–6.

[10] Nagla N. Adaptation accuracy of two different denture base materials for the completely edentulous maxillary arch: an in vitro study. Egypt Dent J. 2017;63:3317–23.

[11] .Lekholm, U., & Zarb, G. A. (1985). Patient selection and preparation. In P.-I. Brånemark, G. A. Zarb, & T. Albrektsson (Eds.), Tissue-Integrated Prostheses: Osseointegration in Clinical Dentistry (pp. 199–209). Quintessence Publishing Co.

[12] Mohamed GF. Clinical evaluation of the efficacy of soft acrylic denture compared to conventional one when restoring severely resorbed edentulous ridge. Cairo Dent J. 2008;24:313–33.

[13] Ahmed Elmorsy A, et al. Do flexible acrylic resin lingual flanges improve retention of mandibular complete dentures? J Int Soc Prev Community Dent. 2015;5(5):365. 26539387 10.4103/2231-0762.165928 PMC4606599

[14] Chandan KR, Tanu M, Ankit M, Rosy R. Comparative evaluation of retention of mandibular special tray by using three different materials for border moulding and final impression. Univ J Dent Sci. 2019;5(2):12–6.

[15] Schwindling FS, Stober T. A comparison of two digital techniques for the fabrication of complete removable dental prostheses: a pilot clinical study. J Prosthet Dent. 2016;116:756–63. 27236597 10.1016/j.prosdent.2016.03.022

[16] Najla C, Yoshiki I, Nada E, Murali S, Frauke M, Sabrina M. Fit and retention of complete denture bases: part II conventional impressions versus digital scans: A clinical controlled crossover study. J Prosthet Dent. 2024;131(4):618–25. 36055812 10.1016/j.prosdent.2022.07.004

[17] Kanakaraj S, Kumar K, Ravichandran R. An update on CAD/CAM removable complete dentures: a review on different techniques and available CAD/CAM denture systems. IJADS. 2021;7(1):491–8.

[18] Sabrina M, Yoshiki I, Nada EO, Murali S, Frauke M, Najla C. Fit and retention of complete denture bases: part I conventional versus CAD-CAM methods: A clinical controlled crossover study. J Prosthet Dent. 2024;131(4):611–7. 36116950 10.1016/j.prosdent.2022.07.006

[19] Jurado C, Sayed M, Fu C, et al. Computer-Aided design and Computer-Aided manufacturing (CAD/CAM) complete dentures for atrophic alveolar ridges: workflow combining conventional and novel techniques. Cureus. 2022;14(1):e21093. 35165553 10.7759/cureus.21093 PMC8830393

[20] Kouveliotis G, Tasopoulos T, Ioannis Karoussis I, Silva NR, Zoidis P. Complete denture digital workflow: combining basic principles with a CAD-CAM approach. J Prosthet Dent. 2022;127(4):550–5. 33549339 10.1016/j.prosdent.2020.12.024

[21] Nadica J, Gordana K, Edvard J. Complete dentures fabricated with CAD/CAM technology and a traditional clinical recording method. Maced J Med Sci. 2017;5(6):785–9. 10.3889/oamjms.2017.169 PMC5661720 29104691

[22] Wang C, Shi YF, Xie PJ, Wu JH. Accuracy of digital complete dentures: a systematic review of in vitro studies. J Prosthet Dent. 2021. 10.1016/j.prosdent.2020.01.004. 32115218 10.1016/j.prosdent.2020.01.004

[23] Hamouda IM, Faramay AMG. Aged flexural properties of vertex thermosens versus conventional denture base material for one years water storage. Austin Dent. 2018;5:1101.

[24] Hsu CY, Yang TC, Wang TM, Lin LD. Effects of fabrication techniques on denture base adaptation: an in vitro study. J Prosthet Dent. 2020;124:740–7. 32448642 10.1016/j.prosdent.2020.02.012

[25] Kalberer N, Mehl A, Schimmel M, Müller F, Srinivasan M. CAD-CAM milled versus rapidly prototyped (3D-printed) complete dentures: an in vitro evaluation of trueness. J Prosthet Dent. 2019;121:637–43. 30711292 10.1016/j.prosdent.2018.09.001

[26] Balch JH, Smith PD, Martin AM, Cagna DR. Reinforcement of a mandibular complete denture with internal metal framework. J Prosthet Dent. 2013;109:202–5. 23522371 10.1016/S0022-3913(13)60045-1

[27] Priyanka T, Ashish P, Arvind KS, Shruti Sh, Pradeep KP. Metal reinforced complete denture: a case report. J Adv Med Dent Sci Res. 2018;6(10):73–7.

[28] Prachi G, Jyotsna S, Maya D, Milind K. Metal reinforced single complete denture -a case report. Int J Med Oral Res. 2018;6(2):38–40.

